# The Use of Digital Twins in Finite Element for the Study of Induction Motors Faults

**DOI:** 10.3390/s21237833

**Published:** 2021-11-25

**Authors:** Tiago Drummond Lopes, Adroaldo Raizer, Wilson Valente Júnior

**Affiliations:** 1 Department of Electrical Engineering, Federal Institute of Santa Catarina, Av. Ver. Abrahão João Francisco, 3899, Itajaí 88307-303, Brazil; wilson.valente@ifsc.edu.br; 2 Department of Electrical Engineering, Federal University of Santa Catarina, R. Roberto Sampaio Gonzaga, s/n, Florianópolis 88040-900, Brazil; raizer@eel.ufsc.br

**Keywords:** condition monitoring, digital twin, fault diagnosis, finite element method, non-destructive testing methods, simulation 3D models, three-phase induction motor

## Abstract

Induction motors play a key role in the industrial sector. Thus, the correct diagnosis and classification of faults on these machines are important, even in the initial stages of evolution. Such analysis allows for increased productivity, avoids unexpected process interruptions, and prevents damage to machines. Usually, fault diagnosis is carried out by analyzing the characteristic effects caused by the faults. Thus, it is necessary to know and understand the behavior during the operation of the faulty machine. In general, monitoring these characteristics is complex, as it is necessary to acquire signals from the same motor with and without failures for comparison purposes. Whether in an industrial environment or in laboratories, the experimental characterization of failures can become unfeasible for several reasons. Thus, computer simulation of faulty motors digital twins can be an important alternative for failure analysis, especially in large motors. From this perspective, this paper presents and discusses several limitations found in the technical literature that can be minimized with the implementation of digital twins. In addition, a 3D finite element model of an induction motor with broken rotor bars is demonstrated, and motor current signature analysis is used to verify the fault effects. Results are analyzed in the time and frequency domain. Additionally, an artificial neural network of the multilayer perceptron type is used to classify the failure of broken bars in the 3D model rotor.

## 1. Introduction

Among all types of electric motors available on the market, the most popular is the three-phase induction [1,2]. After all, this equipment stands out compared to others for having characteristics such as: high efficiency, simple construction, robustness, high starting torque, low maintenance, and convenient power–volume ratio [3]. It is estimated that around 40% of the world electricity production is consumed by these machines, which are the largest energy consumers in the industrial sector (80%) [4,5].

Thus, three-phase induction motors (TIM) are considered reliable equipment that do not fail frequently. However, it commonly operates exposed to unfavorable environmental conditions, such as the presence of humidity and dust. Still other factors, such as power quality problems and mechanical overload, corroborate the faults appearance in these motors [6].

Incipient faults at TIM can be originated mechanically or electrically. Faults considered mechanical are responsible for approximately 40% to 50% of the operation interruption and are generally related to bearings, bearing wear, or still eccentricity [7]. The most common electrical faults are problems in the stator winding, which represent about 37% of faults in TIM, and the rotor bars breakage, which accounts for 10% of these occurrences [8]. These faults affect the motors reliable operation, which is strategically very important for the essential provision services and for industrial processes flow continuity.

In the industrial environment, the sectors in charge of preserving the good functioning of electric motors are under continuous pressure to reduce maintenance costs and prevent unscheduled downtime. Therefore, the need for fault diagnosis in electrical machines is crucial to avoid performance degradation, malfunction, and even irreversible damage. This occurs especially in the case of large machines, where the costs and responsibilities involved are much higher.

On that subject, it is possible to observe that, for some time now, several researchers around the world have been dedicated to the failures study in TIM, their effects, causes, and methodologies for their characterization. Regardless of the type, origin, or malfunction cause, it is always possible to observe changes in the machine functional characteristics and its operation. The most used characteristics for fault diagnosis include unbalanced stator currents [9] and voltages [10], oscillations and torque reduction [11], overheating [12], excessive vibration [13], audible noise [14], distortion of flux [15,16], and electromagnetic field [17].

It is worth noting that each fault diagnosis strategy has its advantages and limitations, some of which are considered invasive, as it is necessary to stop the machine operation for the installation of sensors. Due to this inconvenience, non-invasive fault diagnosis techniques, such as the motor current signature analysis (MCSA), have been highlighted in the technical literature [18,19,20,21,22,23], according to the notes in the next section.

### 1.1. Fault Diagnosis Studies Review at TIM

As mentioned before, it is observed that early fault detection is a challenge for a series of research works that scope is focused on the development of different analysis tools and data acquisition methods. Thus, to detect induction motor faults, different techniques are used, such as MCSA, thermal and vibration analysis, etc. Additionally, it is worth noting that to obtain a reliable fault diagnosis, the TIM analysis requires a substantial amount of data acquisition to characterize the machine functionality and the characteristics caused by faults in their most diverse severity degrees.

Various signal processing tools and intelligent systems can be successfully used in different motor operating conditions for fault diagnosis [9,10,11,12,13,14,15,16,17,18,19,20,21]. The state-of-the-art review previously presented reveals that most of the troubleshooting techniques addressed demand the installation of sensors inside (invasive techniques) or around the motor to obtain the parameter to be analyzed. This need may require operation interruption, which, in most cases, is not acceptable. Additionally, the cost of sensors and their installation can be so high that it becomes impracticable.

Among the fault diagnosis strategies mentioned, motor current signature analysis is the most used methodology due to its different advantages, discussed in the following.

### 1.2. Motor Current Signature Analysis (MCSA)

MCSA uses the specific frequency components of the stator current spectrum, which is called fault signatures, to detect faults in the TIM [18].

This technique allows that the monitoring, detection, and diagnosis of motor conditions to be carried out during machine operation. Stator current can be obtained through telemetry measuring devices or through protection devices that provide the measured value.

Additionally, there is the possibility to measure the current remotely and to transmit it online, which means that the data for the current analysis technique is accessible during the motor operation entire period in various supervisory systems. Furthermore, MCSA shows itself as a versatile tool that can include parametric analysis methods, non-parametric methods, and high resolution or subspace methods.

Several works in the current literature report the successful use of the MCSA technique for diagnosing motor failures. In [22], the Shannon entropy index and a fuzzy logic system are proposed to diagnose stator short-circuit faults. The proposed methodology is based on the MCSA, using the current monitored during the steady state of the induction motor and considering different severity levels and different load conditions.

The research [23] brings forward a study of the use of transient current to analyze the beginning of short-circuits between the induction motor stator windings turns. The proposed methodology extracts the second component of motor starting current signals transient envelope by principal component analysis.

In the study [24], a real-time detection scheme of incipient short-circuit failure between stator of induction machines turns powered by frequency inverter is presented. An analysis based on discrete wavelet transform (DWT) is performed on the stator current and support vector machine (SVM) learning algorithm is used for the accurate incipient fault classification.

The paper [25] proposes an effective method of fault diagnosis using Teager–Kaiser energy operator (TKEO) to detect broken rotor bars faults based on motor current signal analysis. TKEO is applied to remove the main component of motor current for accurate extraction of fault characteristics, especially for an induction motor operating with low load and low slip.

A broken rotor bars fault classification model based on the stator current of induction motors analysis is depicted in [26]. In this study, the principal component analysis (PCA) method is used to reduce the signal size and to extract typical fault characteristics.

In [27], a technique to perform the MCSA with a reduced leakage of the fundamental component is presented. This technique is based on rectified current signal spectral analysis. Its spectrum is shown to contain the same fault harmonics as the original current signal spectrum but at a much lower frequency and free from leakage of fundamental components.

The MCSA technique is used in [28] to monitor induction motor bearings. To improve the monitoring performance, it is proposed to take advantage of more information available in the current spectrum, incorporating the amplitude of a significant number of sidebands around the first eleven harmonics, exponentially increasing the number of fault signatures.

Thus, most mechanical and electrical faults that can arise in an induction motor are detectable by current analysis [22,23,24,25,26,27,28]. For these reasons, the current signature has become a practical parameter for detecting squirrel cage motor faults.

### 1.3. Signal Processing for Fault Analysis and Its Limitations

Normally, the direct use of current signals in the time domain is not convenient in fault diagnosis. They have a low signal-to-noise ratio and problems such as electromagnetic interference. Thus, the use of data processing methods and/or intelligent algorithms based on artificial neural networks (ANN) can be an alternative [29]. Neural networks of type multilayer perceptron (MLP) have a prominent position among the possible tools used in fault diagnosis. This is mainly due to perceptions that this tool is able not only to identify the incipient failure, but also to estimate its severity. [30]. This network architecture can automatically learn, based on experience, the primary representation of the raw signal without requiring complex mathematical models, which makes its implementation simple and accessible.

However, the effectiveness of these networks is directly linked to the availability of a comprehensive database, containing signals from healthy and faulty motors operating under different load conditions and fault severity [4].

In this context, it is highlighted that the creation of these databases is complex and can often become a major obstacle. It is necessary to acquire signals from the same motor with and without faults in different operating conditions. The sampling of these signals in an industrial environment or in laboratories can become impractical for several reasons, such as the need to carry out destructive tests, high financial cost of installing the test bench, availability of equipment, sensors, and motors, and the high demand of time and human resources to carry out all the necessary tests. Due to the aforementioned difficulties, it is possible to observe that there are some databases that have been formed for years, with a slow and difficult evolution, which continue to be insufficient [31]. In most cases, it is impossible to examine all types and conditions of failures physically and experimentally.

These factors are especially aggravated for higher horsepower motors. In addition to being more expensive, it is often not possible to replace them quickly, due to high costs, difficulty in maintenance, and because they are often manufactured to order and for a specific application [3].

A possible alternative solution for this inconvenience is the creation of a dataset through computer simulation of faulty machine models. These models can prove so accurate in representing the characteristics and effects of faults in real motors that they are called digital twins, as discussed below.

### 1.4. Digital Twins Modeling in Finite Elements

The application of computer simulation methods to robustly model faulty motors (e.g., digital twins) in order to create parameterized databases to identify the evolution of faults in real machines, is a promising technique. In this regard, the finite element method (FEM) stands out among the various computational modeling techniques, showing itself as an adequate tool for the purpose. The main motivation for using FEM is that this simulation type offers consistent results, considering the non-linear BH characteristics of the rotor and stator core, the skin effect, the variation of constitutive parameters as a temperature function, and the material dispersivity characteristics. These characteristics make FEM a relevant tool for the design and robust modeling of electrical machines and therefore the analysis and modeling of their faults for the diagnostic study of these induction motor faults [2].

It is noteworthy that, in recent years, some authors have reported success in using the FEM for modeling some types of faults. In [32,33], FEM is used together with other tools to identify broken rotor bar faults. The work [32] evaluates the performance of a strongly coupled two-dimensional (2D) magnetomechanical approach, available directly in COMSOL commercial finite element analysis (FEA) software. This software is used for simulating an induction machine in direct starting, with healthy and broken bar states. The simulation time interval is sufficient to allow the detailed study of the variable frequency components. Results produce, in addition to the usual electrical and magnetic quantities, vibration components induced in the stator.

The paper [33] presents a method for detecting broken rotor bar in a squirrel cage induction motor. The method is based on the stator transient current signal spectral analysis during countercurrent braking (CCB). This type of broken rotor bar fault diagnosis is independent of load conditions and can be performed even for an unloaded motor. The existence of spectral components in the CCB signal is proven with the symmetric components theory. The method is verified through FEA simulations.

The works [34,35,36,37] use different motor quantities and FEA to diagnose stator faults. More specifically, a faulty induction motor modeling technique is depicted in [34]. Using ANSYS Maxwell software, the FEM allows for detailed simulation (two to two turns resolution) of insulation degradation in a stator slot. DWT is used to provide detection of stator windings insulation deterioration.

The methodology presented in [35] uses the flux in the air gap of induction motors for detection of turn–turn failure and faulty region identification. Some search coils (SCs) are used to measure magnetic flux in various regions of the machine air gap. The induced voltages in the SCs are used to assess the level of flux distribution symmetry along the inner stator circumference. The proposed method is verified through FEM simulations performed in Ansoft Maxwell software.

In [36], an offline method of short-circuit fault diagnosis between induction motors stator turns is presented. The proposed method is based on impedance unbalance in the stationary d-q plane. To show impedance unbalance, an induction motor model is presented with a circuit loop and fault resistance. Using the fast fourier transform (FFT) applied to the impedance components in the d-q plane, the second order impedance magnitudes are obtained. From these magnitudes, short-circuit fault and faulty phase can be detected. To verify the proposed method, FEA is presented.

The effect of short-circuit fault location between stator turns on the parameters of the squirrel cage induction machine model is studied in [37]. Two investigations are conducted. The first is a FEA of four cases of fault location with equal severity in a machine phase. The second is a theoretical and mathematical analysis, which the fault is modeled by a step-down autotransformer in the faulty circuit. The results obtained with the FEM show that different locations affect the motor parameters.

Amid a wide-ranging literature review on the use of FEM in the induction motor faults diagnosis, the authors Liang, Ali, and Zhang [38] highlight that the method may offer signals for the analysis of faulty motors.

### 1.5. Digital Twins and Neural Networks for Improving Fault Diagnosis Process

As mentioned in Section 1.3, up until now, it is possible to observe a considerable effort to obtain data about TIM fault in their different degrees of severity, especially because all employed data usually require destructive and time expensive tests [39,40,41,42,43,44]. The main interest regarding this paper, and an almost unexplored issue in this research line, is the use of FEM combined with neural networks to improve fault diagnosis algorithms. Digital twins can provide substantial data gains in the training of these networks and a significant contribution to the study of useful life prediction of induction motors.

In this regard, it is possible to highlight three main contributions:

- **Robust 3D FEM Modeling and MCSA:** In order to provide diagnostic evaluation of the fault behavior, the use of simplified two-dimensional models does not consider important characteristics, such as the effects of the stator coil heads, inter-bar current, and the skew influence of the rotor bars that can considerably change the MCSA (motor current signature analysis). It is important to mention that MCSA is considered a crucial information for fault diagnosis analysis and to this research. This issue justifies the use of robust 3D models (digital twins), unlike typical motor designs simulations, where 2D simulations are normally acceptable.
- **Automated Fault Diagnosis Algorithm:** After obtaining the digital twin simulation results, the MCSA from simulation model is used as an input for an automated fault diagnosis algorithm, based on a multilayer perceptron (artificial neural networks). The algorithm training was set up in real motor data, based in extensive test bench results [39,40,41,42,43,44]. The main goal is to observe if the algorithm is able to adequately classify if the motor is heathy or faulty.
- **Digital Twin to provide network learning:** During the current research, the algorithm training was usually based on an extensive number of destructive test and measurements to provide sufficient data for network learning. Further evaluations must use digital twin simulations in order to deliver sufficient data to virtual network learning. To our understanding, the use of measurement and simulation data can be combined to provide synergy and a considerable improvement to the neural network performance.

## 2. Numerical Modeling of TIM with Faults

An induction motor model, with typical parameters, was implemented in order to verify the characteristics of broken rotor bars faults. As these faults cause asymmetry in the motor, naturally, it can be imagined that it is needed to implement three-dimensional (3D) FEM to be able to represent the faulty machines. Thus, a 3D model of a three-phase induction motor was created with the following parameters: power of 3800 W (5 hp), Y supply voltage of 380 V with 60 Hz frequency, four poles, nominal torque of 21 Nm, and rated speed of 1727 rpm. The parameters used to create the model are based on an IP55 three-phase induction motor from the manufacturer WEG model W22. In addition, this motor was chosen so that the simulation results were later classified by an artificial neural network previously trained for these motor configurations.

### 2.1. Simulations

The tool used to develop and simulate the models uses FEA. Through a magnetic transient solver, the 3D magnetic fields problems in the time domain are solved. The stator windings of motor are supplied by voltage sources that vary in function of time. Rotational motion effects are included in the simulation [45].

As a boundary condition, the master/slave was chosen, since the model has symmetry cuts. In this condition the magnetic field at the slave boundary is forced to match the magnitude and direction (or the negative of the direction) of the magnetic field at the master boundary. Symmetry planes are selected in periodic structures, where the magnetic field is oblique to the boundary. The model stator conductors are considered stranded, so they do not have eddy currents and are considered very fine filaments.

### 2.2. Simulations Hardware

The simulations were carried out using the structure available at the Electromagnetism and Electromagnetic Compatibility Laboratory of the Federal University of Santa Catarina—MagLab/UFSC. It can be used for numerical calculations of simulations that require a greater computational effort, which is the case of FEM 3D models. This is a high-performance computing (HPC) structure with an Intel processor Xeon Gold 6126 with eight cores, 2.6 GHz, and 128 GB RAM memory. The use of HPC generates a simulation time reduction in 3D FEM models, which enables the use of this technique to represent motor failures.

### 2.3. Solver Parameters and Simulation Settings

For both simulations, healthy motor, and broken motor with rotor bars fault, the following parameters were adjusted: stop time of 1.1 s and time step of 290 µs. These parameters guarantee a sampling of approximately 57 points per cycle, considering a frequency of 60 Hz. The time step and the number of segments in rotational band should be synchronized according to rotor speed. Thus, at each calculation step, the rotor moves in exactly one band segment. This procedure aims to reduce calculation noise and improve the results accuracy.

### 2.4. Model Geometry and Mesh Details

**Figure 1:**
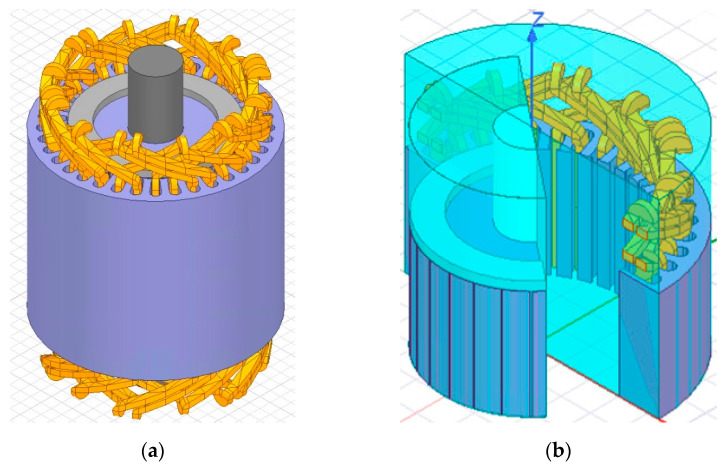
(**a**) 3D induction motor model; (**b**) details of stator, rotational band, and boundary conditions.

Figure 1 shows the 3D FEM model simulated in this work. On the left side, it is possible to observe geometric modeling details of the core and stator windings; on the right side, the rotational band; and the boundary conditions are also presented.

The geometry data considered for both models are presented in Table 1. Using these parameters, the model was designed, and its parts were characterized with constitutive materials. It is noteworthy that the non-linearities found in the materials were considered, which effectively affect the machines behavior. The modeling takes into account the stacking factor of the stator and rotor core lamination. Additionally, the model presented in this study considers the effects of the motor operating temperature on the rotor short-circuit bars and rings.

Even with the availability of HPC to carry out the simulations, 3D FEM are very complex and require great computational effort, demanding long periods to complete the calculations. Thus, to reduce the simulation time, the model was simplified into two symmetry axes in the XY and XZ planes, so only 1/4 of the model was simulated. Comparative tests have been performed with complete model, and it was verified that the results in steady state are equivalent. The maximum error is 3.77%, and the average error is 1.52% for the healthy model. In addition, for the broken bars fault model, the maximum error is 3.93%, and the average error is 1.55%. These results show that the simplifying assumptions do not compromise the model accuracy and are a good trade off to reduce simulation time.

**Figure 2:**
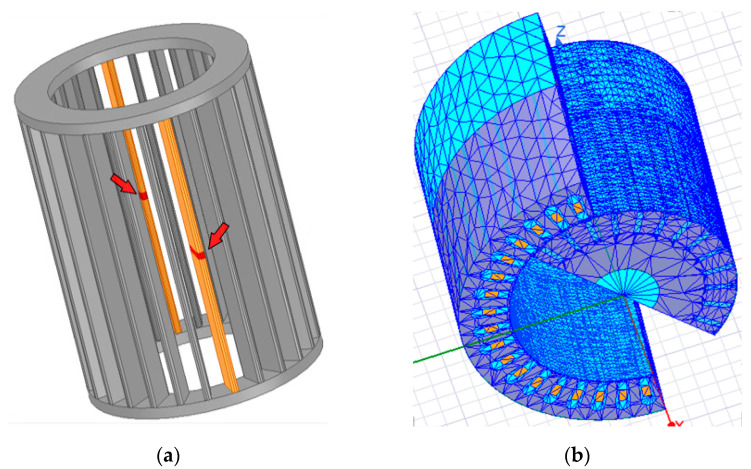
(**a**) Position and detail of broken bars in rotor cage; (**b**) finite element mesh.

The bars breakage was reproduced by inserting vacuum objects in the center of the bars, as can be seen in Figure 2a. It is noteworthy, in the figure in question, that the bars in orange color are the bars with faults, and the red elements indicated by the arrows represent the breaks.

These vacuum elements interrupt current flowing through the bars, reproducing the bar breakage effects on a real rotor. Additionally, the insertion these elements allows specific mesh features to be added to create a denser mesh in place. In this way, it is possible to obtain more accurate field calculations and better represent the effects caused by the fault. Additionally, to obtain a finer mesh in the air gap, vacuum cylinders can be added to serve as a reference in the mesh creation process. Finite element mesh details can be seen in Figure 2b. The generated meshes have approximately 945 thousand (healthy motor) and 990 thousand tetrahedrons (broken bars). A significant difference in the number of elements between the meshes can be observed, mainly because the model with faults needs more elements at the bars breaking points.

## 3. Results

This section presents some results obtained from the simulation of the 3D finite element model implemented according to the characteristics described in the previous section. Results were obtained from the motor simulation operating under two conditions, namely: healthy and fault of 1-1 broken diametrically opposite rotor bars.

### 3.1. Time Domain Analysis

**Figure 3:**
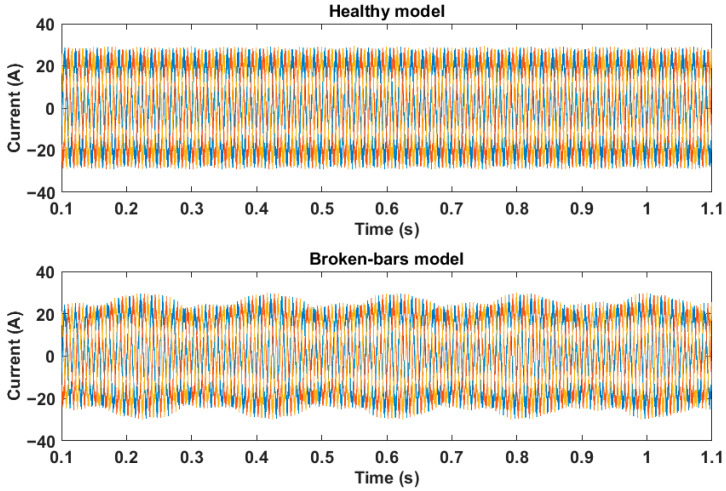
Currents of the models with and without fault in the time domain.

Initially, the fault characteristics of broken bars are analyzed in the time domain. For that, Figure 3 presents the three-phase current simulation of the healthy and 1-1 broken-bar motor models plotted in the time domain.

From the observation of the curves in Figure 3, it is possible to notice changes in the motor current signal in the time domain when there are broken bars. The faulty model curves show deformations and oscillations that were not observed in the currents of the healthy model. These oscillations are typical of broken bar faults and are reflected in the motor speed and torque signal. Thus, as discussed above, without the application of any analysis technique, it is difficult to confirm the effects caused by faults in time-domain signals. This fact demonstrates the need to use signal processing methods.

### 3.2. Frequency Domain Analysis

**Figure 4:**
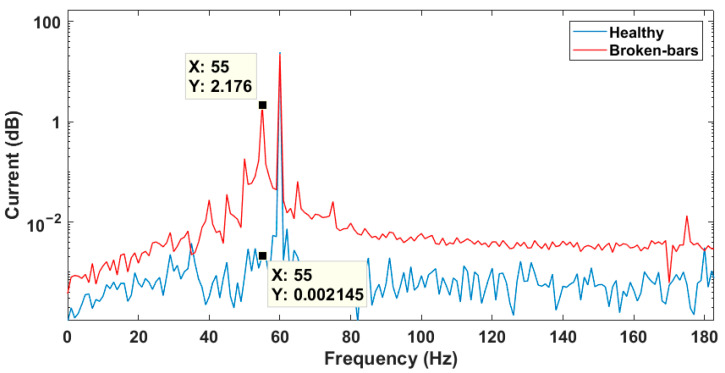
Frequency spectrum of healthy and faulty motor currents.

FFT of the signals was calculated to observe the current frequency response. It is noteworthy that, for the calculation of the Fourier transform, the motor start transient was discarded. Only the steady state signal was used. Thus, it is possible to use the MCSA to characterize the failure signatures. The currents in the frequency domain of the same phase, from the simulation of healthy TIM and with rotor failure, are shown in Figure 4. It is verified that, as described in [10,15,18,22,23,29,33,38,46,47,48], the left sideband amplitude of the main frequency component of the faulty model was higher compared to the healthy one.

Considering that the slip of the motor in question for such operating conditions is 4.28%, the increase in amplitude occurred in the region of the rotor fault frequency. More specifically, the amplitude at the 55 Hz frequency had a significant increase, showing itself as a broken-bar fault signature.

Thus, it was possible to verify the typical broken-bar faults signature in the stator current signal, which have already been previously reported by many papers in technical literature [10,15,18,22,23,29,33,38,46,47,48].

### 3.3. Motor Classification with Artificial Neural Networks (ANN)

In order to complement the results analysis obtained using the 3D FEM model, the simulated current response was submitted to an artificial neural network. This network is a multilayer perceptron type, and it has been trained with healthy motor current signals and with broken bars in the time domain. Signals used as ANN inputs are represented by vectors with 25 elements that represent the three-phase current waveforms. Then, the normalization of that signal is performed by the maximum value and then the data is presented to the network. Recently, studies such as [39,40,41,42,43,44] used the aforementioned methodology and obtained promising results that support the use of the procedure.

#### 3.3.1. Network Training and Validation

For the ANN training, data from the healthy motor and with broken bars operating directly connected to the grid, with different voltage imbalance conditions in the supply and with variation in the load torque, were used. Considering these conditions, there is a total of 220 samples, 110 for the healthy motor and 110 for the motor with broken bars.

The pre-processed data were divided into two groups, training and validation. In the tests, the k-fold cross-validation method was used with 10 subsets for training and validation. The original dataset is randomly divided into k subsets. One of the subsets is sorted to test and validate the network model, and the remaining k−1 subsets are used to train the network. The cross-validation process is repeated k times with each of the k subsets. After completing the cross-validation process, the errors accuracy is calculated providing a reliable measure of the classifier model capacity.

In the MLP network training process, backpropagation was used as the learning algorithm. The learning rate parameter was set to 0.3, the momentum term is 0.2, and the number of epochs for convergence is equal to 500. These parameters were defined empirically considering the MLP classifier performance as a number function of inputs and neurons in the intermediate layer.

Different network configurations were tested by varying the hidden layer neurons number, with 10, 13, 16, 22, and 25 neurons. The configuration with 19 neurons in the hidden layer showed the best results. Thus, the network used in this work has 25 inputs empirically defined with a single hidden layer. It is composed of 19 artificial neurons, which use hyperbolic tangent as activation function, and 2 neurons in the output layer that consider a linear activation function.

Table 2 shows the parameters and accuracy of this network. It is possible to observe that the network presented 100% accuracy in the cross validation, correctly classifying all samples of the healthy motor and with broken bars.

Kappa statistic can be defined as a measure of association used to describe or test the degree of agreement, or reliability and accuracy, in the classification. This index is calculated based on the data in the confusion matrix, more specifically, on the amounts of false positives and false negatives presented in the classification results. This measure has a maximum value of “1”, so the network has full agreement [49].

#### 3.3.2. Digital Twin Diagnosis Process

After the validation of the network, it can be used to classify the results obtained from the 3D FEM model presented in this work. For this, current signals in the time domain are used. A half-period of each of the three-phase currents is randomly selected and discretized with 25 points, and the amplitude of each point is considered. Then, each half cycle is normalized by the peak value.

**Figure 5:**
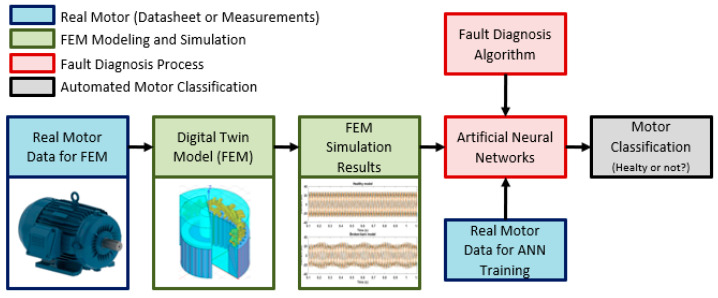
Block diagram for digital twin fault diagnosis.

Thus, there are two data samples, one referring to the healthy model and the other to the model with broken bars. These samples were presented to the neural network to be classified into two possible groups, healthy or faulty. The complete block diagram developed to automatically classify and diagnose the digital twin performance is presented in Figure 5. It takes into account not only the FEM modeling and simulation, but also the fault diagnosis process and ANN outputs.

Table 3 presents the parameters and accuracy of ANN in classifying simulation data from the 3D FEM model.

It can be seen in the results presented in Table 3 that the MLP network managed to correctly classify the two conditions presented. It should be noted that the data were unprecedented for the network; that is, the samples tested did not participate in the ANN training process. Even so, the network achieved 100% accuracy with full agreement (kappa = 1). Thus, it can be verified that the FEM 3-D model presented is capable of representing broken rotor bars failures. These results suggest the possibility of using digital twins for the formation of motor failure databases through computational simulation.

## 4. Discussion

Due to the wide applicability of the induction motor in the most diverse sectors of the economy and the need for its reliable operation, the early failures diagnosis is essential to avoid unexpected stops and losses. In this context, the FEM has stood out as an alternative to traditional methods for creating databases with faulty motor signals.

This research indicates, through a literature review, the possibility of using the FEM to create digital twins of faulty motors in order to diagnose failures. In this regard, it presented the implementation of 3D models healthy and with faults of a three-phase induction motor. Healthy and faulty motor simulations of 1-1 broken diametrically opposite bars were performed.

Through the results obtained from these models, the presence of fault signatures of broken rotor bars in the stator current signal was verified. For this purpose, time and frequency domain analyzes of the 3D FEM model current signals were performed. An increase in the amplitude of the broken-bar fault frequency can be observed, located in the left sideband of the fundamental frequency of the current signal. In addition, an artificial neural network of the multilayer perceptron type is used to classify faults with broken bars in the motor through the 3D FEM model current signals.

Thus, the results achieved indicate the possibility of creating accurate 3D models of motors (digital twins) in order to study and understand the characteristics of faults through current, torque, speed, electromagnetic field, and flux. These quantities can be used to create faulty motor databases and later be analyzed by data processing methods and/or intelligent algorithms for fault diagnosis.

Furthermore, it should be noted that the simulation strategy presented makes it possible to analyze the most diverse types and severity of failures, which are often not practicable experimentally due to the need for destructive tests, high financial cost with bench, equipment, sensors, and motors and need for availability of time and human resources.

In summary, the development of digital twins (precise models) of machines would be very useful for examining the operational characteristics of faulty machines. The use of digital twins reduces the need for destructive testing, as well as being used to validate new techniques for fault diagnosis or training and condition monitoring systems testing based on artificial intelligence, such as artificial neural networks. Thus, the high costs associated with machines, experimental benches, and destructive tests would be greatly reduced, especially in the case of large machines with failures that can hardly be tested in the laboratory. The economy and all the impact of the use of digital twins could be more observed in industry and in power generation, since this is where the biggest machines are found.

## 5. Conclusions

The results presented in this research indicate the possibility of creating faulty induction motor digital twins. It was possible to verify the typical characteristics and failures signatures through time and frequency domain analyses. An artificial neural network of the multilayer perceptron type correctly classified faults of broken bars in the rotor of the 3D FEM model. So, in this paper, we prove that digital data results can be recognizable by artificial neural networks using automated diagnosis algorithms in order to adequately classify a healthy or broken-bar motor model. Thus, the authors suggest that the research advance towards the use of digital twins in FEM to create a parametrized database of healthy and faulty motors, not only for different levels of severity, but also for different load conditions. Thus, it will be possible to train fault diagnosis systems with simulation data in order to identify faults in real motors, featuring an alternative non-destructive training method.

## Figures and Tables

**Table 1:** Model geometry data.

Item	Stator	Rotor
Outer Diameter	175 mm	120.3 mm
Inner Diameter	121 mm	38 mm
Length	150 mm	150 mm
Number of Slots	36	26
End Ring Width	-	6 mm
End Ring Height	-	17 mm

**Table 2:** Parameters and accuracy of the neural network.

Parameter	Result
Inputs	25
Accuracy (%)	100
Building time (s)	1.36
Kappa statistic	1

**Table 3:** ANN Parameters and accuracy for classification testing of the 3D FEM Model.

Parameter	Result
Inputs	25
Accuracy (%)	100
Testing time (s)	0.001
Kappa statistic	1
